# The diagnostic value of mpMRI and PSMA-targeted PET/CT in radiorecurrent prostate cancer: a narrative review

**DOI:** 10.3389/fonc.2026.1782344

**Published:** 2026-04-21

**Authors:** Umberto Merani, Alessio Venturi, Giorgio Calleris, Alessandro Dematteis, Giuseppe Carlo Iorio, Alessandro Marquis, Alberto Sasia, Giancarlo Marra, Paolo Gontero, Marco Oderda

**Affiliations:** 1 Department of Surgical Sciences, Division of Urology, Molinette Hospital, University of Turin, Turin, Italy; 2 Department of Oncology, Radiation Oncology Unit, Molinette Hospital, University of Turin, Turin, Italy

**Keywords:** imaging, MRI, multiparametric, PET/CT, PSMA, radiorecurrent

## Abstract

**Background:**

Biochemical recurrence after radiotherapy is common and clinically challenging in prostate cancer (PCa), as accurate restaging is required to identify patients eligible for local salvage therapy and distinguish them from those requiring systemic or metastasis-directed treatment. This review evaluates the diagnostic value of multiparametric MRI (mpMRI) and prostate-specific membrane antigen–targeted PET/CT (PSMA-targeted PET/CT) for restaging radiorecurrent prostate cancer.

**Methods:**

A narrative review was conducted using PubMed and Scopus. Original English-language studies published within the last 10 years were included if they reported the diagnostic performance of mpMRI, PSMA-targeted PET/CT, or combined imaging in patients with suspected radiorecurrent prostate cancer, using histopathology as the reference standard. Evidence was synthesized with particular attention to intraprostatic recurrence, extraprostatic disease, and imaging performance after brachytherapy.

**Results:**

Ten studies (4 prospective and 6 retrospective) met the inclusion criteria. mpMRI demonstrated heterogeneous sensitivity for intraprostatic recurrence with moderate-to-high specificity (64–87%), and frequently underestimated multifocal disease, particularly in the post-brachytherapy setting. PSMA-targeted PET/CT showed high sensitivity for intraprostatic recurrence (up to ~89%) and superior detection of nodal and distant metastases, although very small or low–PSMA-expressing intraprostatic lesions may remain undetected. The combination of mpMRI and PSMA-targeted PET/CT provided the highest diagnostic confidence: concordant findings achieved a positive predictive value of 97.6%, supporting improved patient selection for salvage treatment strategies.

**Conclusions:**

mpMRI and PSMA-targeted PET/CT provide complementary diagnostic information rather than being interchangeable modalities. A multimodal imaging approach improves restaging accuracy in radiorecurrent prostate cancer and may better guide biopsy targeting and selection of candidates for salvage therapy. Nevertheless, histological confirmation remains mandatory before local salvage treatment.

## 1 Introduction

Prostate cancer (PCa) is the most common malignancy among men in Europe and a leading contributor to cancer burden worldwide (1). Radiotherapy (RT) is widely employed as a curative modality, but up to one quarter of patients eventually develop biochemical recurrence (BCR), which may reflect persistent or recurrent disease at local, regional, or distant sites (1, 2). If we estimate that in Europe around 100,000 RTs were performed to treat primary PCa in 2022, we can expect a yearly rate of 25,000 radiorecurrent cases, making radiorecurrent PCa the 4th most common genito-urinary malignancy in men (2). Accurate localization of recurrence is mandatory to select appropriate management, including salvage radical prostatectomy (sRP) or focal salvage options such as high-intensity focused ultrasound (HIFU), salvage brachytherapy and re-irradiation with stereotactic body radiotherapy (SBRT) (3, 4).

Modern imaging has reshaped the work-up of radiorecurrent PCa. Multiparametric magnetic resonance imaging (mpMRI) and prostate-specific membrane antigen positron emission tomography/computed tomography (PSMA-targeted PET/CT) are essential in restaging these patients, combining morphological and functional imaging. mpMRI provides superior anatomical resolution for local therapy planning, whereas PSMA-targeted PET/CT offers good sensitivity for detecting local and distant disease (1). Despite these complementary strengths, significant variability in diagnostic accuracy persists across studies, raising important questions about how reliably each technique characterizes the true burden of recurrence. This narrative review therefore aims to synthesize the diagnostic performance of mpMRI and PSMA-targeted PET/CT in radiorecurrent PCa, highlight their respective limitations, and outline practical imaging-guided pathways to support salvage treatment decision-making.

## 2 Methods

A comprehensive literature search was conducted using the PubMed and Scopus databases. The following Medical Subject Headings (MeSH) terms and keywords were used in different combinations: “prostate cancer”, “radiorecurrent”, “imaging”, “multiparametric MRI”, and “PSMA PET”. Only original studies published in English within the last 10 years were considered. Reviews, meta-analyses, editorials, commentaries, case reports, studies without histopathological confirmation, and studies not specifically focused on diagnostic accuracy in the radiorecurrent setting were excluded. Reference lists of relevant articles were also screened to identify additional eligible studies.

Studies were eligible if they (1) evaluated mpMRI and/or PSMA-targeted PET/CT for detection of radiorecurrent prostate cancer after radiotherapy, (2) reported histopathological confirmation of recurrence, and (3) provided diagnostic performance metrics such as sensitivity, specificity, or predictive values.

**Figure 1:**
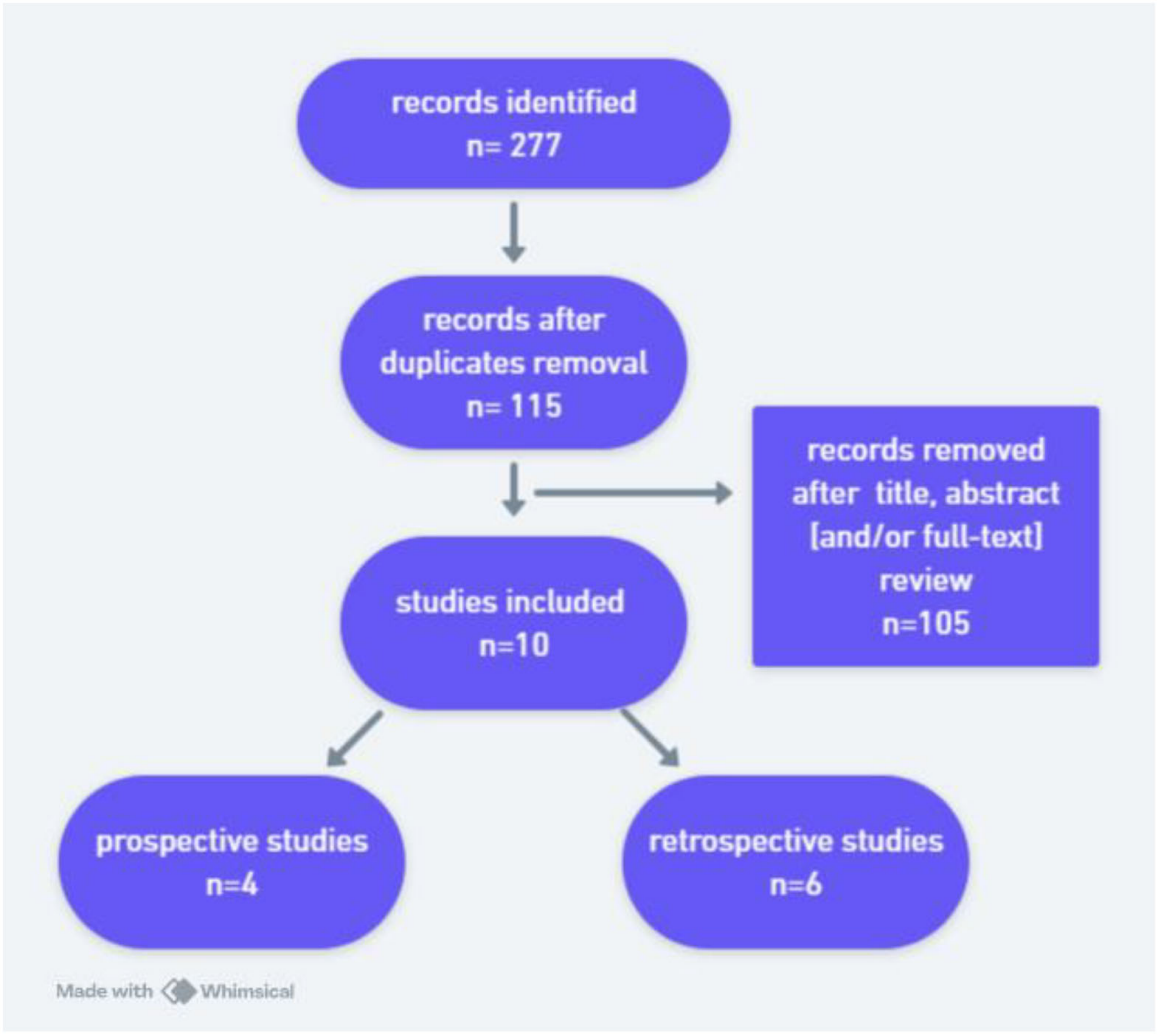
study flow diagram.

The search yielded 277 records (**Figure 1**). After removal of duplicates, 115 studies underwent title and abstract screening. Following full-text evaluation, 10 original studies met the inclusion criteria and were included in this narrative review, comprising 4 prospective and 6 retrospective studies. Given the limited number of high-quality original studies available in this specific clinical context, both prospective and retrospective studies were included without formal weighting. This approach reflects the narrative nature of the review and aims to provide a clinically meaningful synthesis of the most relevant and methodologically robust evidence, rather than a quantitative meta-analysis. Radiopharmaceutical nomenclature was standardized according to the International Consensus Radiochemistry Nomenclature Guidelines (5). Although a structured literature search and predefined inclusion criteria were applied, this study was designed as a narrative review rather than a systematic review, as no formal risk of bias assessment or quantitative meta-analysis was performed due to the limited number and heterogeneity of the available studies. Main characteristics of included studies are shown in **Table 1**.

**Table 1:** Studies evaluating diagnostic accuracy of mpMRI and/or PSMA PET/CT in the radiorecurrent setting.

Author, year	Study design	Patients enrolled	Imaging modality	Level of analysis for intraprostatic recurrence	Reference pathology	PSA at imaging (ng/mL)	Prior ADT
Abd-Alazeez et al., 2015 (6)	Retrospective single-centre diagnostic accuracy study	37	mpMRI (T2W, multi-b DWI, ADC maps, DCE) evaluated with sequential-read paradigm	Sector-based (ROI-based)	Template prostate mapping (TPM) biopsy at 5-mm grid as gold standard	4.5 (IQR 3–7.3)	27/37 (72,9%)
Zattoni et al., 2016 (7)	Retrospective single-centre cohort.	19	3T mpMRI with endorectal coil (T2-weighted, DWI, DCE)	Whole-gland (per-patient)	Salvage radical prostatectomy with extended pelvic lymph-node dissection	4.9 (IQR 3.0-6.8)	–
Kanoun et al., 2017 (8)	Retrospective	32	mpMRI, [¹^8^F]F-choline PET/CT, combined	Hemi-gland (right vs left prostate)	3D-Transperineal mapping biopsy	2.92 (IQR 1.39-8.71)	8/32 25%
Dinis Fernandes et al., 2019 (9)	Retrospective MRI–pathology correlation study	19	3T mpMRI (T2-weighted, DWI, DCE; quantitative voxel-wise analysis)	Sector-based/voxel-wise	Step-section whole-mount prostatectomy with MRI–histology co-registration	5.4 (IQR 3.4-6.1)	–
Kowa et al., 2021 (10)	Retrospective single-centre salvage RP series after RT	24	3T mpMRI (PI-RADS v2 multiparametric protocol)	Whole-gland (per-patient)	Whole-mount radical prostatectomy (tumour localisation and pT stage)	3.2 (IQR 2.3–5.2)	–
Shah et al., 2022 (FORECAST MRI arm) (11)	Prospective diagnostic accuracy study	155	3T mpMRI (T2-weighted, DWI, DCE)	Whole-gland (per-patient)	Transperineal template-mapping biopsy (5-mm grid) + MRI-targeted biopsies	4 (IQR 2–6)	79% (reported in overall cohort, n=181) **
Rasing et al., 2022 (12)	Prospective cohort evaluating combined imaging in candidates for focal salvage after RT	41	Combined mpMRI + [^68^Ga]Ga-PSMA-11 PET/CT for intraprostatic recurrence localisation	Lesion-based (imaging-positive lesions only; PPV-based)	Targeted prostate biopsies (cognitive or MRI/PSMA-TRUS fusion) confirming biopsy-proven intraprostatic recurrence	4.6 (IQR 2.7–7.8)	–
Light et al., 2023 (FORECAST MRI arm) (13)	Prospective paired-cohort diagnostic accuracy study	144	3T mpMRI (T2-weighted, DWI, DCE) for intraprostatic recurrence after RT	Whole-gland (per-patient)	Transperineal template-mapping biopsy (systematic + targeted cores) used as gold standard	3.80 (IQR 2.40–6.17)	none 19%, neo-adjuvant 58%, adjuvant 23%**
Light et al., 2024 (14)	Retrospective single-centre diagnostic accuracy study	35	[^68^Ga]Ga-PSMA-11 PET/CT and mpMRI evaluated separately and in combination for hemigland recurrence	Hemi-gland	Transperineal prostate biopsy confirming presence/absence of intraprostatic recurrence (hemigland-based comparison)	3.80 (IQR 2.60–5.53)	none 11%, neo-adjuvant 27%, adjuvant 30% both 33%
Venkatesulu et al., 2025 (F-SHARP secondary analysis) (15)	Secondary analysis of a prospective phase I/II salvage HDR brachytherapy trial	62	mpMRI, ^18F-fluciclovine PET/CT and ^18F-DCFPyL PSMA PET/CT were used for identification and delineation of intraprostatic radiorecurrent lesions (IPRLs).	Whole-gland (per-patient)	Systematic and targeted prostate biopsies defining biopsy-proven intraprostatic radiorecurrence	4.7 (range 2.2–20.3)	–

mpMRI multiparametric magnetic resonance imaging; PET, positron emission tomography; PSMA prostate-specific membrane antigen. EBRT, external beam radiotherapy; ERC, endorectal coil;DWI, diffusion-weighted imaging; ADC, apparent diffusion coefficient; DCE, dynamic contrast-enhanced imaging; TPM, template prostate mapping biopsy; RP, radical prostatectomy; HDR, high-dose-rate; IPRR, intraprostatic radiorecurrent lesion. **ADT within 6 months of imaging was excluded.

## 3 Evidence synthesis

The results in terms of sensitivity, specificity, and diagnostic accuracy of mpMRI and/or PSMA-targeted PET/CT in the recurrent setting are summarized in **Table 2**. Across the included studies, median PSA levels at the time of imaging ranged from 2.9 to 5.4 ng/mL. Reporting of prior androgen deprivation therapy (ADT) was heterogeneous; when available, rates of prior ADT ranged from 25% to 79%, and some prospective studies excluded patients receiving recent ADT. This variability in biochemical burden and hormonal status may partly account for differences in reported diagnostic performance.

**Table 2:** Diagnostic performance of mpMRI and/or PSMA-targeted PET/CT in the recurrent setting.

Study	Imaging	Setting	Sensitivity	Specificity	Accuracy
Abd-Alazeez et al., 2015 (6)	mpMRI	Intraprostatic recurrence	~78%*	~70%*	AUC 0.80–0.84
Zattoni et al., 2016 (7)	mpMRI	T3 disease Nodal recurrence	EPE** 50–71% SVI 62–77% PLNM 60%	EPE** 80-100% SVI 66.6% PLNM 86-93%	/
Kanoun et al., 2017 (8)***	mpMRI + [¹^8^F]F-choline PET/CT	Intraprostatic recurrence	mpMRI 32% PET-choline 34% MpMRI+PET-choline 43%	mpMRI 87% PET-choline 87% MpMRI+PET-choline 83%	mpMRI 64% PET-choline 65% MpMRI+PET-choline 66%
Dinis Fernandes et al., 2019 (9)	mpMRI	Intraprostatic recurrence****	/	/	Qualitative (underestimation)
Kowa et al., 2021 (10)	mpMRI	Intraprostatic recurrence T3 disease	IPR^ 64% EPE 31% SVI 45%	IPR 94% EPE 100% SVI 96%	/
Shah et al., 2022 (FORECAST MRI arm) (11)	mpMRI	Intraprostatic recurrence ^^	Likert ≥3 94% Likert ≥4 81%	Likert ≥3 18% Likert ≥4 88%	
Rasing et al., 2022 (12) ^^^	mpMRI + [^68^Ga]Ga-PSMA-11 PET/CT	Intraprostatic recurrence	/	/	PPV 97,6%
Light et al., 2023 (FORECAST MRI arm) (13)	mpMRI	Intraprostatic recurrence	95-98%	19-21%	/
Light et al., 2024 (14)	mpMRI + [^68^Ga]Ga-PSMA-11 PET/CT	Intraprostatic recurrence	mpMRI 89% PET-PSMA 72% MpMRI+PET-PSMA 98%	mpMRI 67% PET-PSMA 64% MpMRI+PET-PSMA 45%	
Venkatesulu et al., 2025 (F-SHARP secondary analysis) (15)	mpMRI, ^18F-fluciclovine PET/CT and ^18F-DCFPyL PSMA PET/CT	Intraprostatic recurrence	mpMRI 91,8% PET-PSMA 85,5%	/	/

mpMRI multiparametric magnetic resonance imaging; PET, positron emission tomography; PSMA prostate-specific membrane antigen. PPV, positive predictive value; NPV, negative predictive value; AUC, area under the receiver operating characteristic curve; *Values derived from final mpMRI read; where multiple readers were reported, mean values are presented. **EPE (extraprostatic extension), SVI (seminal vesicle invasion),PLNM (pelvic linfonodes metastasis)*** Data derived from segment-based analysis using 3D transperineal mapping biopsy as reference standard.**** This study did not report conventional diagnostic accuracy metrics but provided qualitative evidence on tumour detection and delineation. ^ IPR (intraprostatic recurrence). ^^ For the FORECAST study, mpMRI performance is reported according to different positivity thresholds (Likert ≥3 vs ≥4), illustrating the expected trade-off between sensitivity and specificity. ^^^ In Rasing et al., diagnostic performance metrics were limited to positive predictive value, as biopsies were performed only in imaging-positive lesions.

### 3.1 mpMRI: performance and pitfalls

Across contemporary original studies included in this review, mpMRI demonstrated heterogeneous diagnostic performance in the radiorecurrent setting (**Table 2**). Studies using template-mapping biopsy or whole-mount prostatectomy as the reference standard reported a wide range of sensitivity and specificity for the detection of intraprostatic recurrence, reflecting differences in study design and level of analysis (per-patient, hemigland, or sector-based) (6, 8, 11, 13). Direct MRI–histology correlation studies consistently showed that mpMRI frequently underestimates the true extent of recurrent disease, even when the dominant index lesion is identified (7, 9, 10). This limitation was particularly evident in multifocal recurrence, where hemigland- or segment-based analyses reported reduced diagnostic accuracy when mpMRI was used as a standalone modality (8).

Diagnostic performance was further challenged in the post-brachytherapy setting, where radiation-induced changes and susceptibility artefacts contribute to reduced lesion conspicuity, leading to missed biopsy-proven lesions and limited reliability for confident lesion exclusion (9, 15). Overall, while mpMRI provides accurate anatomical localisation of visible lesions, its standalone reliability for comprehensive disease mapping remains limited in radiorecurrent prostate cancer.

### 3.2 PSMA-targeted PET/CT: diagnostic value

Among the studies included in this review, the most robust evidence on PSMA-targeted PET/CT derives from the cohorts reported by Light et al. and Venkatesulu et al. (14, 15). Across these studies, PSMA-targeted PET/CT demonstrated high diagnostic performance for the detection of intraprostatic recurrence, with sensitivity comparable to or higher than mpMRI depending on the study design and level of analysis (**Table 2**). Beyond local recurrence, [^68^Ga]Ga-PSMA-11 PET/CT enabled the identification of nodal and distant metastatic disease that was frequently occult on mpMRI, supporting its role in systemic restaging in the radiorecurrent setting (14, 15). This whole-body detection capability allows more accurate stratification of patients with isolated intraprostatic recurrence versus those with extra-prostatic disease. Overall, [^68^Ga]Ga-PSMA-11 PET/CT provides a more comprehensive assessment of disease burden compared with mpMRI alone, while mpMRI retains a complementary role for detailed anatomical localisation of intraprostatic lesions (14, 15).

Moreover, detection rates of PSMA-targeted PET/CT have been shown to increase with rising PSA levels and shorter PSA doubling time in the broader biochemical recurrence setting, reflecting its sensitivity to tumour burden and biological aggressiveness (16). However, robust evidence supporting this relationship specifically in the strictly radiorecurrent setting remains limited.

### 3.3 Combined imaging: mpMRI and PSMA-targeted or [¹^8^F]F-choline PET/CT

Across the original studies included in this review, combined imaging with mpMRI and PET-based techniques demonstrated higher diagnostic confidence compared with either modality alone, although the magnitude of improvement varied according to tracer type and level of analysis (**Table 2**).

In the prospective cohort reported by Rasing et al., concordant mpMRI and [^68^Ga]Ga-PSMA-11 PET/CT findings for intraprostatic recurrence were almost always confirmed by targeted biopsy, resulting in a very high positive predictive value (12). As biopsies were performed only in imaging-positive lesions, conventional diagnostic performance metrics such as sensitivity and specificity could not be assessed in this study. In contrast, studies using non-PSMA tracers reported a more limited incremental benefit. In the series by Kanoun et al., the combination of mpMRI with [¹^8^F]F-choline PET/CT resulted in only a modest improvement in diagnostic accuracy compared with either modality alone (8).

More recent evidence from Light et al. demonstrated that combining mpMRI and [^68^Ga]Ga-PSMA-11 PET/CT substantially increased sensitivity and negative predictive value for intraprostatic recurrence on a hemigland basis, albeit at the expense of reduced specificity compared with standalone imaging (14).

**Figure 2:**
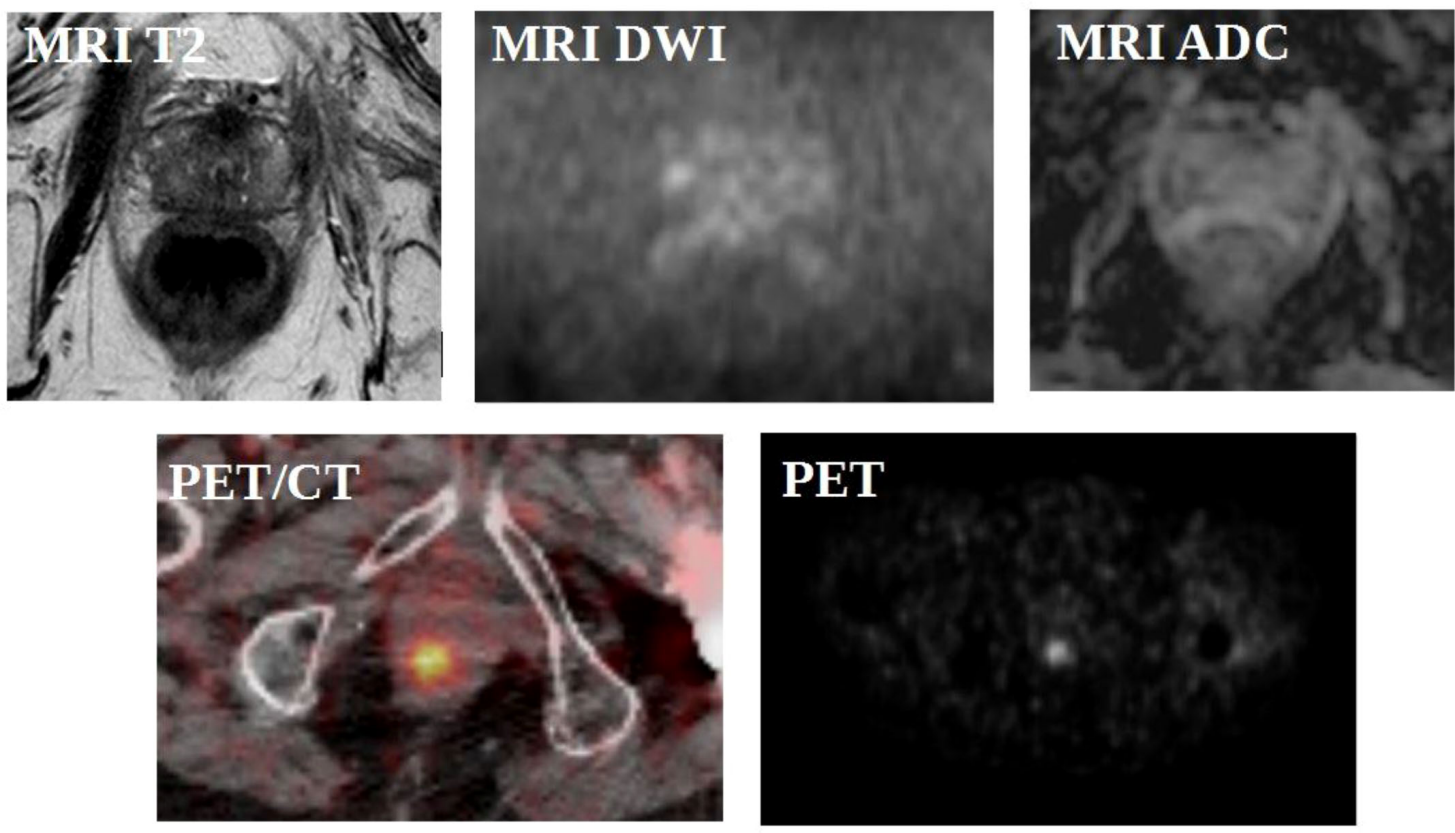
Representative illustration of intraprostatic radiorecurrent prostate cancer detected by mpMRI and [^68^Ga]Ga-PSMA-11 PET/CT of a patient followed in Molinette Hospital, adapted from published examples in the literature.

A representative example illustrating the complementary role of mpMRI and PSMA PET/CT in the localization of intraprostatic radiorecurrent disease is shown in **Figure 2**.

### 3.4 Post-brachytherapy recurrence

After brachytherapy, mpMRI shows substantially reduced performance for intraprostatic recurrence, with high rates of missed biopsy-proven lesions and underestimation of disease extent (9). This limitation is particularly relevant in the setting of low-dose-rate (LDR) brachytherapy with permanent seed implantation, where susceptibility artefacts and distortion of prostatic anatomy may significantly impair lesion conspicuity. In contrast, in high-dose-rate (HDR) brachytherapy, where no permanent seeds are implanted, these artefacts are less pronounced, although post-radiation changes may still affect image interpretation (9).

In contrast, PSMA-targeted PET/CT maintained robust diagnostic performance after brachytherapy. In the cohort reported by Rasing et al., [^68^Ga]Ga-PSMA-11 PET/CT detected the majority of biopsy-confirmed intraprostatic recurrences that were not identified on mpMRI (12). In addition, [¹^8^F]F-fluciclovine and [¹^8^F]F-DCFPyL PSMA PET/CT more frequently identified nodal and distant metastatic disease in patients with post-brachytherapy recurrence, reflecting the higher prevalence of extra-prostatic disease in this population (15). When mpMRI and [^68^Ga]Ga-PSMA-11 PET/CT findings were concordantly positive, combined imaging showed very high reliability for confirming intraprostatic recurrence, as reflected by a positive predictive value approaching 98% (12).

## 4 Discussion

Restaging patients with biochemical recurrence after radiotherapy remains a complex and often fragmented diagnostic process. The evidence synthesized from the original studies included in this review confirms that neither multiparametric MRI (mpMRI) nor PSMA-targeted PET/CT alone provides a complete or sufficiently reliable assessment of the true burden of radiorecurrent prostate cancer. Rather than being interchangeable, these modalities provide complementary diagnostic information, and their intrinsic limitations have direct implications for treatment selection, risk stratification, and the feasibility of salvage interventions.

Across contemporary MRI studies, mpMRI demonstrates substantial variability in diagnostic performance in the post-radiotherapy setting, with wide ranges of sensitivity and specificity reported across original series (6–11, 13). Direct MRI–histology correlation studies consistently show that mpMRI underestimates tumour extent, even when the dominant index lesion is identified (7, 9, 10). In particular, multifocal and satellite lesions frequently escape MRI detection, resulting in incomplete disease characterisation when mpMRI is used in isolation. These findings are not study-specific but reflect intrinsic limitations of MRI interpretation in irradiated prostates, as further supported by the meta-analysis by Zapala et al., which demonstrated poor pooled sensitivity despite preserved specificity for both local and nodal staging before salvage radical prostatectomy (17).

MRI performance deteriorates further after brachytherapy, where radiation-induced fibrosis, distortion of prostatic anatomy, and seed-related susceptibility artefacts reduce lesion conspicuity. In this context, the risk of false-negative imaging becomes clinically relevant. Fernandes et al. reported false-negative rates approaching 28%, underscoring the limited reliability of mpMRI for confidently excluding recurrence after brachytherapy (9). These shortcomings may result in inadequate focal-therapy targeting, underestimation of disease burden, or inappropriate exclusion from salvage surgery. In contrast PSMA-targeted PET/CT offers high sensitivity and improved detection of extra-prostatic disease, with performance that may be comparable to or higher than mpMRI depending on the clinical setting and study design. In the cohort reported by Light et al., [^68^Ga]Ga-PSMA-11 PET/CT outperformed mpMRI in sensitivity and negative predictive value for intraprostatic recurrence, while maintaining robust overall diagnostic accuracy (14). Venkatesulu et al. similarly demonstrated that ^18F-fluciclovine and ^18F-DCFPyL PSMA PET/CT maintains reliable performance after brachytherapy, identifying the majority of biopsy-confirmed lesions missed by mpMRI (15). These findings are consistent with broader evidence showing that PSMA-targeted PET/CT provides superior detection of nodal and distant metastases compared with anatomical imaging, thereby improving systemic restaging. In addition, several prospective and multicentre studies conducted in broader biochemical recurrence cohorts further support the high clinical impact of PSMA-based imaging in the post-radiotherapy setting. Liu et al. demonstrated that ^18F-DCFPyL PSMA PET/CT identified extraprostatic disease in a substantial proportion of patients with biochemical recurrence after radiotherapy, leading to changes in clinical management in nearly half of cases (18). Similarly, the LOCATE trial have shown that advanced PET tracers significantly improve detection rates at relatively low PSA levels, frequently revealing oligometastatic or previously unrecognized nodal disease (19). More recently, Evangelista et al. reported high detection rates even below the Phoenix biochemical threshold, underscoring the sensitivity of PSMA-targeted PET/CT in early radiorecurrence (20). Although these studies were not systematically validated against whole-mount pathology, they consistently highlight the substantial incremental value of molecular imaging in real-world restaging scenarios.

Nevertheless, PSMA-targeted PET/CT is not without limitations. Very small intraprostatic lesions, diffuse microscopic disease, or tumours with low PSMA expression may remain undetected, and PET lacks the fine anatomical resolution required for precise focal therapy planning. As a result, PSMA-targeted PET/CT alone cannot fully replace high-resolution anatomical imaging when detailed intraprostatic mapping is required.

Importantly, the clinical indication for advanced molecular imaging must be interpreted within the broader therapeutic context. According to current guideline recommendations (1), PET-based imaging in the setting of biochemical recurrence after radiotherapy should primarily be performed in patients who are potential candidates for curative loco-regional salvage treatments, given the morbidity associated with salvage interventions.

The complementary strengths of mpMRI and PSMA-targeted PET/CT become most evident when the two techniques are combined. Multiple original studies demonstrate that both modalities tend to underestimate disease burden when used independently (8, 12, 14). In the prospective cohort reported by Rasing et al., concordant mpMRI and [^68^Ga]Ga-PSMA-11 PET/CT findings were confirmed by targeted biopsy in nearly all cases, resulting in a positive predictive value of 97.6%, indicating that concordant positivity reliably reflects true tumour recurrence (12). Kanoun et al. observed a modest but consistent improvement in diagnostic accuracy when mpMRI was combined with [¹^8^F]F-choline PET/CT, compared with either modality alone (8). Earlier PET tracers such as [¹^8^F]F-choline and [¹^8^F]F-fluciclovine have historically been used for restaging biochemical recurrence after primary treatment. However, their diagnostic performance is generally inferior to PSMA-targeted radiotracers, particularly at low PSA levels. For this reason, contemporary guidelines increasingly recommend PSMA-based PET imaging as the preferred modality for restaging prostate cancer recurrence, while choline or fluciclovine PET/CT are mainly considered when PSMA imaging is unavailable (21).

More recently, Light et al. showed that combining mpMRI and [^68^Ga]Ga-PSMA-11 PET/CT substantially increased sensitivity and negative predictive value for intraprostatic recurrence, albeit at the expense of reduced specificity, further supporting the role of multimodal imaging in reducing false-negative findings (14).

Beyond imaging performance, clinical context remains critical. PSA level and PSA kinetics play a central role in guiding the choice and interpretation of imaging modalities in men with suspected radiorecurrent disease. PSMA-targeted PET/CT detection rates have been shown to increase with rising PSA levels and shorter PSA doubling time in patients with biochemical recurrence, reflecting its sensitivity to tumour burden and biological aggressiveness (16). Conversely, mpMRI retains particular value for precise anatomical delineation of focal intraprostatic recurrence and local treatment planning, especially in low-volume disease (22). Importantly, histological confirmation with targeted and systematic mapping remains mandatory before local salvage treatments, irrespective of imaging findings (1).

Emerging technologies may further optimize multimodal restaging strategies. Hybrid PET/MRI systems offer the potential to integrate molecular sensitivity with superior soft-tissue contrast while reducing radiation exposure, although prospective validation in the radiorecurrent setting remains limited. Structured interpretation frameworks, such as the recently validated PRIMARY scoring system, improve reproducibility and inter-reader agreement in [^68^Ga]Ga-PSMA-11 PET/CT interpretation and further support standardized combined imaging approaches (23). Structured interpretation frameworks may further improve reproducibility and diagnostic confidence in the radiorecurrent setting. In addition to the recently validated PRIMARY scoring system for PSMA PET/CT, the PI-RR (Prostate Imaging for Recurrence Reporting) scale has been proposed to standardize mpMRI interpretation after radiotherapy, improving inter-reader agreement and supporting more consistent identification of intraprostatic recurrence (22). In addition, radiomics and artificial intelligence-based tools hold promise for improved lesion detection, segmentation, and individualized risk stratification, although their clinical implementation is currently limited by lack of standardization and external validation.

Finally, advances in PSMA theranostics highlight the expanding role of molecular imaging beyond diagnosis. Trials such as ROADSTER and other studies evaluating Lutetium-177 (Lu-177)-PSMA radioligand therapy underscore the importance of PSMA expression as both a diagnostic and therapeutic biomarker across the continuum of recurrent prostate cancer management (24).

Overall, the accumulated evidence strongly supports a multimodal and integrated imaging approach for radiorecurrent prostate cancer. While mpMRI and PSMA-targeted PET/CT each provide valuable but incomplete information when used alone, their combination offers the most comprehensive framework for restaging, guiding biopsy, and selecting candidates for salvage treatment within a personalized management strategy.

## 5 Conclusions

Restaging radiorecurrent prostate cancer remains challenging, and neither mpMRI nor PSMA-targeted PET/CT alone provides a complete evaluation of disease extent. mpMRI offers excellent anatomical detail but is limited by post-radiation changes, while PSMA-targeted PET/CT provides outstanding systemic detection yet may underestimate intraprostatic multifocality. In clinical practice, the combination of mpMRI and PSMA-targeted PET/CT offers the most reliable assessment, substantially improving diagnostic confidence and reducing the risk of misclassification. This integrated approach has direct implications for patient management: it refines selection for salvage focal or surgical therapies, helps avoid futile interventions, and promotes timely identification of systemic or oligometastatic disease requiring alternative treatment strategies.

## References

[1] Parker C Gillessen S Heidenreich A Horwich A . Prostate cancer: ESMO clinical practice guidelines for diagnosis, treatment and follow-up. Ann Oncol. (2015) 26:v69–77. doi: 10.1093/annonc/mdv222. PMID: 26205393

[2] Pound CR Partin AW Eisenberger MA Chan DW Pearson JD Walsh PC . Natural history of progression after PSA elevation following radical prostatectomy. JAMA. (1999) 281:1591–7. doi: 10.1001/jama.281.17.1591. PMID: 10235151

[3] Valle LF Lehrer EJ Markovic D Elashoff D Levin-Epstein R Karnes RJ . A systematic review and meta-analysis of local salvage therapies after radiotherapy for prostate cancer. Prostate Cancer Prostatic Dis. (2021) 24:573–87. doi: 10.1016/j.eururo.2020.11.010. PMID: 33309278 PMC10262981

[4] Cuccia F Mazzola R Nicosia L Giaj-Levra N Figlia V Ricchetti F . Prostate re-irradiation: Current concerns and future perspectives. Expert Rev Anticancer Ther. (2020) 20:947–56. doi: 10.1080/14737140.2020.1822742. PMID: 32909471

[5] Coenen HH Gee AD Adam M Antoni G Cutler CS Fujibayashi Y . International consensus radiochemistry nomenclature guidelines. Nuklearmedizin. (2018) 57:40–1. doi: 10.1055/s-0038-1636563. PMID: 29536500

[6] Abd-Alazeez M Ramachandran N Dikaios N Ahmed HU Emberton M Kirkham A . Multiparametric MRI for detection of radiorecurrent prostate cancer: added value of apparent diffusion coefficient maps and dynamic contrast-enhanced images. Prostate Cancer Prostatic Dis. (2015) 18:128–36. doi: 10.1038/pcan.2014.55. PMID: 25644248

[7] Zattoni F Kawashima A Morlacco A Davis BJ Nehra AK Mynderse LA . Detection of recurrent prostate cancer after primary radiation therapy: An evaluation of the role of multiparametric 3T magnetic resonance imaging with endorectal coil. Am Soc For Radiat Oncol. (2017) 7:42–9. doi: 10.1016/j.prro.2016.06.003. PMID: 27527896

[8] Kanoun S Walker P Vrigneaud JM Depardon E Barbier V Humbert O . 18F-choline positron emission tomography/ computed tomography and multiparametric magnetic resonance imaging for the detection of early local recurrence of prostate cancer initially treated by radiation therapy: Comparison with systematic 3-dimensional transperineal mapping biopsy. Int J Radiat Oncol Biol Phys. (2017) 97:986–94. doi: 10.1016/j.ijrobp.2016.12.025. PMID: 28333020

[9] Fernandes CD Ghobadi G van der Poel HG de Jong J Heijmink SWTPJ Schoots I . Quantitative 3-T multi-parametric MRI and step-section pathology of recurrent prostate cancer patients after radiation therapy. Eur Radio. (2019) 29:4160–8. doi: 10.1007/s00330-018-5819-y. PMID: 30421016 PMC6610274

[10] Kowa JY Soneji N Sohaib SA Mayer E Hazell S Butterfield N . Detection and staging of radio-recurrent prostate cancer using multiparametric MRI. Br J Radiol. (2021) 94:20201423. doi: 10.1259/bjr.20201423. PMID: 33586998 PMC8010529

[11] Shaha TT Kanthabalanc A Otienoc M Pavloud M Omard R Adeleke S . Magnetic resonance imaging and targeted biopsies compared to transperineal mapping biopsies before focal ablation in localised and metastatic recurrent prostate cancer after radiotherapy. Eur Urol Oncol. (2022) 81:598–605. doi: 10.1016/j.eururo.2022.02.022. PMID: 35370021 PMC9156577

[12] Rasing M van Son M Moerland M de Keizer B Wessels F Jonges T . Value of targeted biopsies and combined PSMA PET/CT and mp-MRIimaging in locally recurrent prostate cancer after primary radiotherapy. Cancers. (2022) 14:781. doi: 10.3390/cancers14030781. PMID: 35159048 PMC8834189

[13] Light A Kanthabalanc A Otienoc M Pavloud M Omard R Adeleke S . The role of multiparametric MRI and MRI–targeted biopsy in the diagnosis of radiorecurrent prostate cancer: An analysis from the FORECAST trial. Eur Urol Oncol. (2024) 85:35–46. doi: 10.1016/j.eururo.2023.09.001. PMID: 37778954

[14] Light A Lazic S Houghton K Bayne M Connor MJ Tam H . Diagnostic performance of 68Ga-PSMA-11 PET/CT versus multiparametric MRI for detection of intraprostatic radiorecurrent prostate cancer. J Nucl Med. (2024) 65:379–85. doi: 10.2967/jnumed.123.266527. PMID: 38212074

[15] Venkatesulu BP Adams W Joel R Ross D Yoo R Quick C . The importance of multiparametric magnetic resonance imaging, positron emission tomography/computed tomography, and biopsy for identifying and delineating the extent of intraprostatic radiorecurrent prostate cancer: A secondary analysis of the F-SHARP clinical trial. Int J Radiat Oncol Biol Phys. (2025) 000:1–6. doi: 10.1016/j.ijrobp.2025.02.042. PMID: 40057285

[16] Perera M Papa N Roberts M Williams M Udovicich C Vela I . Gallium-68 prostate-specific membrane antigen positron emission tomography in advanced prostate cancer-updated diagnostic utility, sensitivity, specificity, and distribution of prostate-specific membrane antigen-avid lesions: A systematic review and meta-analysis. Eur Urol. (2020) 77:403–17. doi: 10.1016/j.eururo.2019.01.049. PMID: 30773328

[17] Zapała P Ślusarczyk A Rajwa P Gandaglia G Zapała Ł Zattoni F . Magnetic resonance imaging (MRI) for local staging before salvage radical prostatectomy: A meta-analysis. World J Urol. (2023) 41:1529–39. doi: 10.1007/s00345-023-04383-2. PMID: 37019997 PMC10188391

[18] Liu W Zukotynski K Emmett L Chung HT Chung P Wolfson R . A prospective study of 18F-DCFPyL PSMA PET/CT restaging in recurrent prostate cancer following primary external beam radiotherapy or brachytherapy. Int J Radiat Oncol Biol Phys. (2020) 106:546–55. doi: 10.1016/j.ijrobp.2019.11.001. PMID: 31730876

[19] Adeleke S Latifoltojar A Sidhu H Galazi M Shah TT Clemente J . Localising occult prostate cancer metastasis with advanced imaging techniques (LOCATE trial): A prospective cohort, observational diagnostic accuracy trial investigating whole-body magnetic resonance imaging in radio-recurrent prostate cancer. BMC Med Imaging. (2019) 19:90. doi: 10.1186/s12880-019-0380-y. PMID: 31730466 PMC6858718

[20] Evangelista L Vallone C Guglielmo P Damiani S Jandric J Brignoli A . PSMA PET/CT for the detection of prostate cancer biochemical recurrence after primary radiation therapy: Is it time to review the Phoenix criteria? Eur J Nucl Med Mol Imaging. (2026) 53:3056–63. doi: 10.1007/s00259-025-07699-w. PMID: 41364231

[21] Afshar-Oromieh A Zechmann CM Malcher A Eder M Eisenhut M Linhart HG . Comparison of PET imaging with a (68)Ga-labelled PSMA ligand and (18)F-choline-based PET/CT for the diagnosis of recurrent prostate cancer. Eur J Nucl Med Mol Imaging. (2014) 41:11–20. doi: 10.1007/s00259-013-2525-5. PMID: 24072344 PMC3843747

[22] Panebianco V Villeirs G Weinreb JC Turkbey BI Margolis DJ Richenberg J . Prostate magnetic resonance imaging for local recurrence reporting (PI-RR): International consensus -based guidelines on multiparametric magnetic resonance imaging for prostate cancer recurrence after radiation therapy and radical prostatectomy. Eur Urol Oncol. (2021) 4:868–76. doi: 10.1016/j.euo.2021.01.003. PMID: 33582104

[23] Light A Lazic S Connor MJ Tam H Ahmed HU Shah TT . Validation of the PSMA PRIMARY scoring system and comparison to an E-PSMA Likert system for [68Ga]Ga-PSMA-11 PET/CT interpretation in men with suspected radiorecurrent prostate cancer. Clin Nucl Med. (2026) 51:e1–e10. doi: 10.1097/RLU.0000000000006168. PMID: 41365507 PMC12673892

[24] Mendez LC Dhar A Laidley D Moussa M Gomez JA Chin J . ROADSTER: A phase II trial of 177Lu-PSMA radioligand therapy in prostate cancer. BMC Cancer. (2023) 23:851. doi: 10.1186/s12885-023-10851-0. PMID: 37081426 PMC10116658

